# Low-grade appendiceal mucinous tumor complicated by intussusception

**DOI:** 10.1097/MD.0000000000042540

**Published:** 2025-06-06

**Authors:** Chunzhi Guo, Yan Qu, Hong Liu

**Affiliations:** a Department of Thyroid Surgery, Qingdao Central Hospital, University of Health and Rehabilitation Sciences (Qingdao Central Hospital), Qingdao, Shandong, China; b General Surgery/Department of Gastrointestinal Surgery, The Second Affiliated Hospital of ZunYi Medical University, Zunyi, Guizhou, China

**Keywords:** ileocecal intussusception, low-grade appendiceal mucinous tumor, right hemicolectomy

## Abstract

**Rationale:**

Appendiceal mucinous tumors with intussusception are extremely rare. As primary lesions, malignant tumors often present with atypical clinical symptoms, which increases the likelihood of misdiagnosis.

**Patient concerns:**

A 79-year-old female patient of Miao ethnicity in Guizhou Province, China, was admitted with intermittent paroxysmal abdominal pain for 20 days and abdominal distension for 3 days.

**Diagnoses:**

Computed tomography revealed ileal intussusception in the lower abdomen, suspected to be associated with a tumor.

**Interventions:**

Laparoscopy-assisted right hemicolectomy was performed.

**Outcomes:**

Postoperative histopathology showed a low-grade appendiceal mucinous tumor with mucinous components extending into the muscularis propria and localized calcification. Regular follow-up was recommended.

**Lessons:**

Low-grade appendiceal mucinous tumors are extremely rare and are even less common when complicated by intussusception. Their clinical presentation is nonspecific, which may result in missed diagnosis. Therefore, thorough preoperative evaluation and careful surgical planning are essential for improving prognosis and minimizing the risk of severe complications.

## 1. Introduction

Primary appendiceal tumors, including appendiceal mucinous tumors, are rare and highly heterogeneous. Their atypical clinical presentation poses significant challenges for diagnosis and management.^[1]^ Low-grade appendiceal mucinous neoplasms (LAMNs) and high-grade appendiceal mucinous neoplasms (HAMNs) are characterized by low- and high-grade epithelial features, respectively, and both lack invasive growth.^[2]^ The biological behavior of LAMNs indicates malignant potential. Tumor can infiltrate the deeper layers of the appendiceal wall, extend to adjacent tissues and organs, and disseminate into the peritoneal cavity, resulting in pseudomyxoma peritonei.^[3]^ Therefore, despite their low-grade classification, inadequate management may lead to serious clinical consequences. Molecular analyses have shown that LAMNs may progress to HAMNs due to frequent mutations in KRAS, GNAS, and RNF43.^[4,5]^ The invasive phenotype of HAMNs is associated with mutations in TP53, ATM, and APC genes.^[6,7]^ LAMNs have an insidious onset, lack overt invasive features, and present with nonspecific symptoms, leading to frequent misdiagnosis.^[8,9]^ In this study, we report a rare case of low-grade appendiceal mucinous neoplasm with intussusception in an older female from a Chinese ethnic minority group.

## 2. Case report

### 2.1. Clinical features

A 79-year-old female patient of the Miao ethnic group in Guizhou Province, China, was admitted to the emergency department after experiencing paroxysmal right lower abdominal pain for more than 20 days, which worsened on the day of admission. Mild abdominal distension was also present, without nausea, vomiting, fever, altered bowel habits, or significant weight loss. Physical examination revealed mild tenderness in the right lower abdomen, without rebound tenderness or muscular rigidity. A hard, mobile mass was palpable in the right lower abdomen. Shifting dullness was negative, and bowel sounds were reduced on auscultation. Blood and urine test results were within normal limits, and carbohydrate antigen 19-9 levels were 28.5 U/mL. There was no family history of hereditary disease, genetic disorders, or malignancy.

### 2.2. Imaging examination

**Figure 1.**
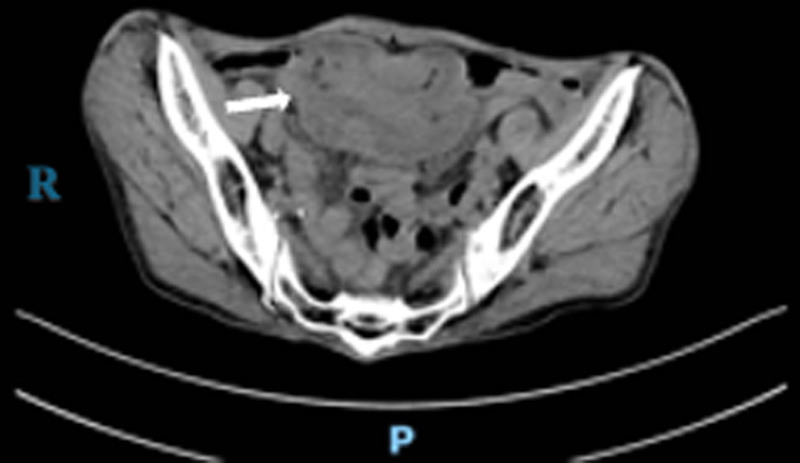
Abdominal computed tomography scan showing ileocecal intussusception (white arrow).

Abdominal computed tomography (CT) revealed ileocecal intussusception, possibly associated with a tumor (Fig. 1).

### 2.3. Surgical treatment and outcomes

**Figure 2.**
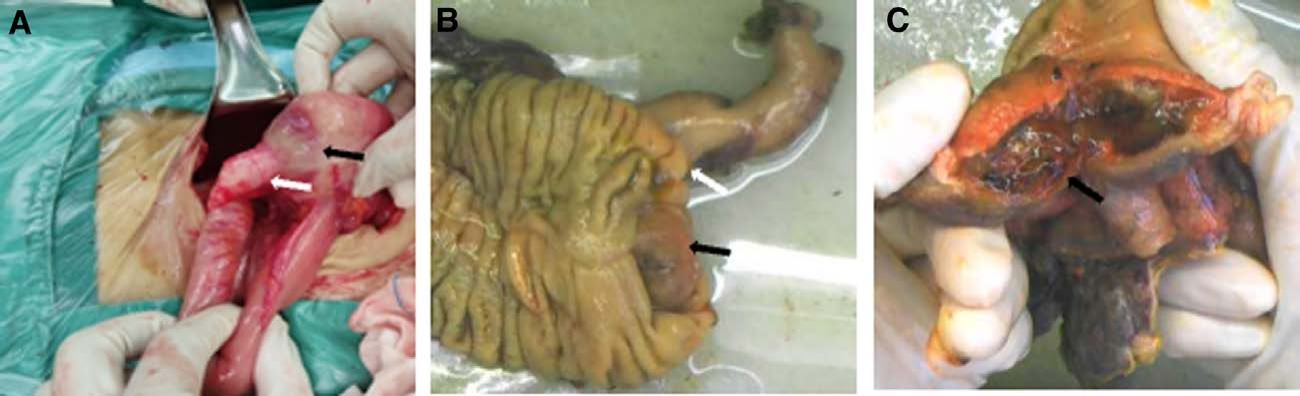
Gross examination. (A, B) Macroscopic observation shows that the tumor originates at the base of the appendix (white arrow) and invades the cecal tissue (black arrow). (C) A large amount of mucinous component is present within the tumor tissue (black arrow).

**Figure 3.**
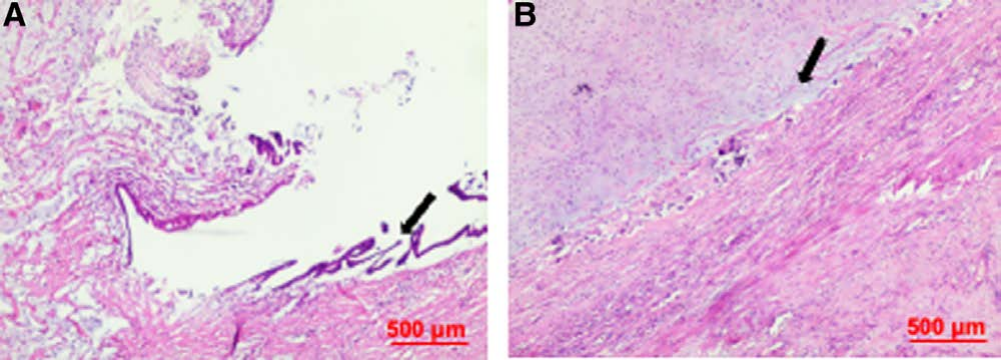
Histopathological features of a low-grade appendiceal mucinous neoplasm (hematoxylin and eosin staining). (A) Tumor cells within the lumen of the appendix (black arrow). (B) Mucinous cells within the cecal tissue (black arrow). Original magnification ×40.

**Figure 4.**
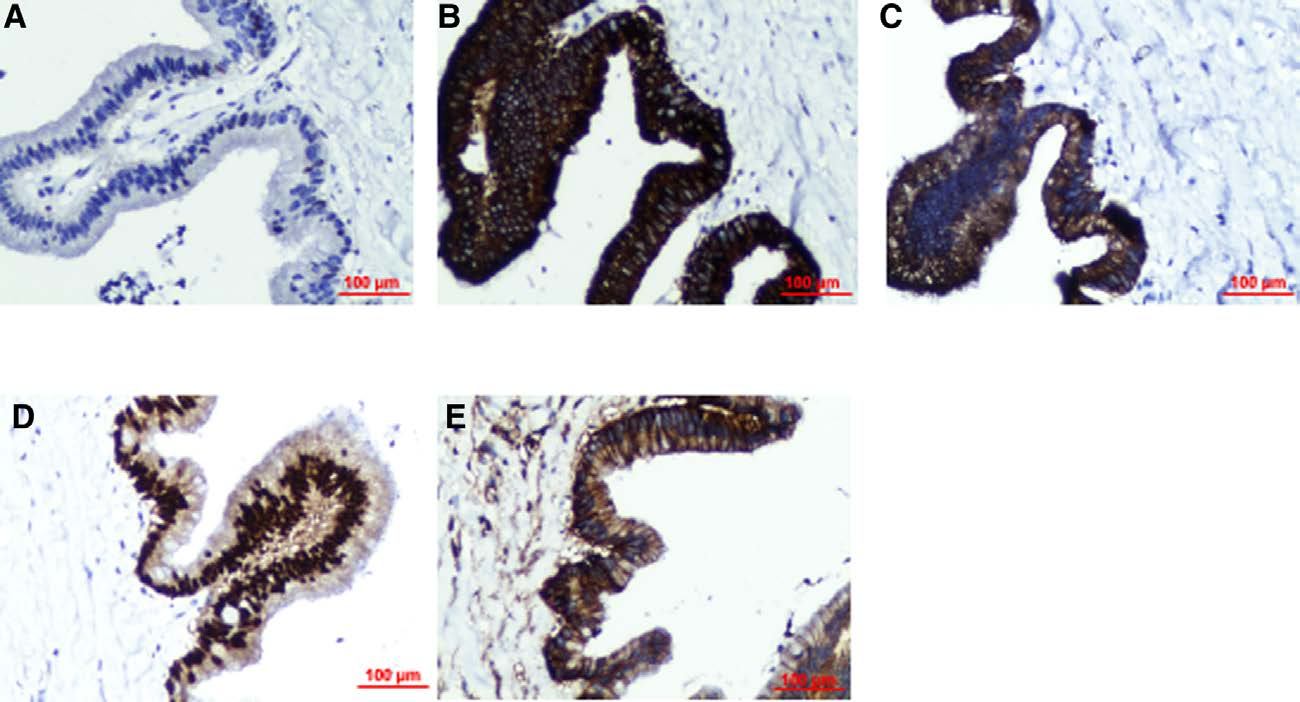
Immunohistochemical staining. (A) P53 wild type, (B) MUC2 positive, (C) CK20 positive, (D) CDX2 positive, (E) CD44 positive. Original magnification × 40. CD44 = cluster of differentiation, CDX2 = caudal-type homeobox 2, CK20 = cytokeratin 20, MUC2 = mucin 2, P53 = tumor protein 53.

A right hemicolectomy with regional lymph node dissection was performed. Intraoperatively, the terminal ileum was found to have invaginated into the cecum, resulting in obstruction. After reduction of the intussuscepted bowel, a tumor mass invading the cecum was identified at the base of the appendix (Fig. 2). Postoperative pathological examination confirmed a low-grade appendiceal mucinous neoplasm, with mucinous components extending into the muscularis propria and localized calcification. No metastases were detected in the mesenteric margins or lymph nodes (pT4b; Fig. 3). The postoperative course was uneventful, and the patient was discharged on postoperative day 9. Long-term follow-up for up to 10 years was recommended. Immunohistochemistry demonstrated wild-type P53, and positivity for mucin 2, cytokeratin 20, caudal-type homeobox 2, and cluster of differentiation 44 (Fig. 4). The Medical Ethics Committee of the Second Affiliated Hospital of Zunyi Medical University approved this report (Approval No.: KYLL-2024-052), and written informed consent was obtained for publication. The data were accessed for research purposes on October 15, 2024. Access to participant data was granted with ethics committee approval. This case report was prepared in accordance with the CARE guideline.^[10]^

## 3. Discussion

According to the American Society of Colon and Rectal Surgeons,^[11]^ the incidence of appendiceal tumors has increased from 0.12 cases per 1,00,000 individuals per year to 0.97 cases per 1,00,000. Therefore, it is essential for surgeons to be familiar with primary appendiceal tumors.

LAMNs with intussusception are extremely rare in clinical practice. In the present case, a 79-year-old female patient from the Miao minority presented with right lower abdominal pain. CT revealed ileocecal intussusception, which was intraoperatively confirmed as LAMN with cecal invasion. This case illustrates the nonspecific clinical features and the high risk of misdiagnosis associated with LAMNs, underscoring the importance of careful physical examination in timely diagnosis. A high index of suspicion for LAMNs should be maintained in patients with atypical abdominal pain,^[12]^ especially when CT findings indicate characteristic ileocecal abnormalities. In such cases, colonoscopy is recommended to clarify the diagnosis and guide clinical management.

The 8th edition of the American Joint Committee on Cancer (AJCC) Cancer Staging Manual^[13]^ substantially revised the Tumor, Node, Metastasis (TNM) staging of LAMNs compared with the 7th edition.^[14]^ Notably, the pT classification and its prognostic implications have been updated.

In the revised TNM staging system, mucinous tumors confined to the mucosa or submucosa without serosal or mesenteric involvement are classified as pTis. Tumors involving the subserosa or appendiceal mesentery are staged as pT3. Tumors breaching the serosal surface are staged as pT4a, while those invading adjacent organs or structures are classified as pT4b.^[15]^ Based on the updated World Health Organization classification of digestive system tumors and the AJCC TNM staging criteria, pathological evaluation and clinical staging were performed for the surgically resected appendiceal specimen in this case. The tumor involved both the appendix and adjacent cecum. Gross examination revealed a gelatinous appearance, and histopathology showed a pushing growth pattern with relatively well-defined borders. The tumor extended to the muscularis propria and contained mucin pools and focal calcification. The lesion involved surrounding bowel segments and invaded the cecum. No metastasis was detected in mesenteric lymph nodes. The final TNM stage was determined as pT4b based on intraoperative and pathological findings. The patient underwent right hemicolectomy with regional lymph node dissection and required long-term follow-up of up to 10 years, including routine imaging.

The updated TNM staging system emphasizes the noninvasive growth characteristics of LAMNs. Based on these criteria, a comprehensive literature review was conducted to reclassify previously reported cases. This facilitates more accurate treatment planning and enables precise prediction of recurrence risk and long-term prognosis. Furthermore, it supports personalized follow-up strategies, helping to prevent both overtreatment and undertreatment and optimize resource allocation.

In the literature review, relevant cases retrieved from PubMed were reclassified using the updated World Health Organization and AJCC staging systems (Table 1). A search using the term “appendiceal mucinous tumor with intussusception” was conducted for studies published between January 2000 and October 2024. A total of 23 articles were retrieved. After excluding 1 review, 1 case of appendiceal endometriosis, and 1 case of mucinous cystadenocarcinoma with intestinal malrotation, 21 cases, including the present case, were included in the analysis. Among the 21 patients, 7 were male and 14 female. Abdominal pain was the primary presenting symptom in 18 cases; 1 case presented with diarrhea, 1 with hematochezia and altered bowel habits, and 1 was asymptomatic, detected during routine physical examination. CT was the initial diagnostic modality in 17 cases, colonoscopy in 2, and ultrasound in 2. Coexisting conditions included neuroendocrine carcinoma (carcinoid) at the appendiceal tip, multiple intestinal hemangiomas, a right-sided follicular-luteal ovarian cyst, and a moderately to poorly differentiated rectal adenocarcinoma. According to the AJCC 8th edition staging manual, 8 cases were classified as pTis and 13 as pT4. The literature suggests that cross-sectional imaging, particularly CT, is highly effective for evaluating appendiceal mucinous tumors. CT is useful for confirming or excluding appendiceal involvement in cases of intussusception and may indicate a more specific diagnosis. Additionally, the male-to-female ratio for appendiceal cysts was noted to be 1:4.^[17]^ Some reports^[36,37]^ have described concurrent ovarian cystadenocarcinoma and appendiceal mucinous tumors. Endometriosis^[38]^ frequently affects the gastrointestinal tract. These observations raise the possibility of a pathological link between female reproductive organs and the appendix, potentially contributing to the higher incidence of appendiceal mucinous tumors in females. Further investigation is warranted to determine whether the incidence varies among women from different ethnic backgrounds.

**Table 1:** Review of case reports of appendiceal mucinous tumor with intussusception from January 2000 to October 2024.

Literature	Sex	Age	Manifestation	Histology	Diagnostic methods	Intussusception location	Treatment
Chan and Tank^[16]^	Female	41	Right-sided abdominal pain and abdominal distension	LAMN pT4b	Colonoscopy	Ileocecal intussusception	Laparoscopic right hemicolectomy
Coulier et al^[17]^	Male	40	Pain in the right upper abdomen and right lower abdomen	LAMN pT4b	Ultrasound	Ileocecocolic intussusception	Ileocecal resection
Butte et al^[18]^	Female	41	Right lower abdominal pain and weight loss	LAMN pTis, Neuroendocrine carcinoma	CT	Ileocecal intussusception	Laparoscopic right hemicolectomy
Hsieh and Lin^[19]^	Female	66	Epigastric pain	LAMN pT4b	CT	Ileocecal intussusception	Laparoscopic right hemicolectomy
Oliphant et al^[20]^	Female	20	Epigastric pain	LAMN pT4a	CT	Ileocecal intussusception	Laparoscopic right hemicolectomy
Yang et al^[21]^	Male	47	Abdominal pain and bloating	LAMN pT4b	CT	Ascending colon intussusception combined with ileocecal intussusception	Laparoscopic right hemicolectomy
Chua et al^[22]^	Female	30	Right lower abdominal pain	LAMN pTis, Multiple intestinal hemangiomas	CT	Transverse colon intussusception combined with ileocecal intussusception	Cecectomy and appendectomy
Park et al^[23]^	Male	69	Diarrhea	LAMN pTis	Colonoscopy	Sigmoid colon intussusception	Laparoscopic appendectomy
Nakamatsu et al^[24]^	Female	43	Right lower abdominal pain	LAMN pT4b	CT	Transverse colon intussusception	Ileocecal resection with D2 lymph node dissection
Rudek et al^[25]^	Male	52	Right lower abdominal pain	LAMN pT4b	CT	Ileocecal intussusception	Ileocecal resection with regional lymph node dissection
Sun et al^[26]^	Female	57	Hematochezia and changes in bowel habits	LAMN pTis, Rectal medium-low differentiated adenocarcinoma	CT	Ileocecal intussusception	ileocecal resection
Waseem et al^[27]^	Female	84	Acute intestinal obstruction	LAMN pT4b	CT	Ileocecocolic intussusception	Laparoscopic right hemicolectomy
Yamaguchi et al^[28]^	Female	32	Abdominal pain and diarrhea	LAMN pT4b	CT	Transverse colon intussusception	Ileocecal resection
Teke et al^[29]^	Female	37	Right lower abdominal pain	LAMN pT4b	CT	Ileocecal intussusception	Ileocecal resection
Zabeirou et al^[30]^	Male	25	Abdominal pain	LAMN pTis	CT	Ileocecocolic intussusception	Laparoscopic right hemicolectomy
Feliu et al^[31]^	Female	57	Right lower abdominal pain	LAMN pT4b	CT	Ileocecal intussusception	Laparoscopic right hemicolectomy
Kishikawa et al^[32]^	Female	57	Right lower abdominal pain	LAMN pTis	CT	Ileocecocolic intussusception	Laparoscopic right hemicolectomy
Okuda et al^[33]^	Male	44	Routine physical examination	LAMN pTis	Ultrasound	Ascending colonic intussusception	Ileocecal resection
Cois et al^[34]^	Female	36	Pain in the mid-abdomen	LAMN pTis, right follicular-lutein ovarian cyst	Ultrasound	Ileocecal intussusception	Ileocecal resection
Wei-Ming et al^[35]^	Male	28	Right lower abdominal pain	LAMN pT4b	CT	Ileocecal intussusception	Laparoscopic right hemicolectomy

CT = computed tomography, LAMN = low-grade appendiceal mucinous neoplasm.

## Author contributions

**Conceptualization:** Hong Liu.

**Data curation:** Yan Qu.

**Formal analysis:** Yan Qu.

**Writing – original draft:** Chunzhi Guo, Hong Liu.

**Writing – review & editing:** Chunzhi Guo, Hong Liu.
